# Reply: Beyond Chronological Age in PCI Decision-Making

**DOI:** 10.1016/j.jacadv.2026.103414

**Published:** 2026-11-06

**Authors:** Yuichi Saito, Yuichiro Mori, Kyohei Yamaji, Shun Kohsaka, Ken Kozuma

**Affiliations:** a Department of Cardiovascular Medicine, Chiba University Hospital, Chiba, Japan; b Department of Health Promotion and Behavioral Sciences, Graduate School of Medicine, Kyoto University, Kyoto, Japan; c Department of Cardiology, Kyoto University, Kyoto, Japan; d Department of Cardiology, Keio University School of Medicine, Tokyo, Japan; e Department of Cardiology, Teikyo University Hospital, Tokyo, Japan

We thank Dr Güner and colleagues for their careful reading of our nationwide analysis of percutaneous coronary intervention (PCI) across age strata.^1^ Their comments give us a welcome opportunity to clarify the intent of the study. Using Japanese nationwide registry data, we showed that the mean age of patients undergoing PCI has steadily increased from 2019 to 2024, with a particularly notable increase in nonagenarians, whereas in-hospital mortality and complications remained higher at advanced age.^1^ Our primary aim was descriptive: to characterize how population aging is being reflected in contemporary PCI practice in one of the world’s most rapidly aging societies.

As Dr Güner and colleagues point out,^2^ geriatric domains such as frailty and cognitive decline should have a critical impact on mortality. An important limitation of the present nationwide registry is the lack of data on geriatric domains. However, chronological age and frailty are not interchangeable. Age is a fixed marker that captures accumulated biological and clinical risk, whereas frailty reflects multidimensional vulnerability and may differ substantially among patients of the same chronological age. Thus, although age is useful for characterizing population-level risk, frailty, cognitive function, functional status, and other geriatric domains may better inform whether an older patient is likely to benefit from PCI.

Importantly, patients in their 90s undergo PCI after substantial clinical selection based not only on conventional comorbidities, but also on such geriatric domains. Because this clinical selection might favor better outcomes in nonagenarians, the poor outcomes observed even under such selection may further highlight the potential influence of underlying geriatric vulnerability. Notably, our results never support withholding PCI solely based on age. Primary PCI in patients with acute myocardial infarction and percutaneous revascularization in those with severe refractory angina may remain valuable procedures regardless of age despite procedural risk. Therefore, shared decision-making that accounts for individual geriatric domains and personal goals of care is particularly important when considering PCI in the oldest-old.

Dr Güner and colleagues also raise concern regarding outcomes among the oldest patients. Although in-hospital mortality among nonagenarians appeared to increase over time in our study, this trend was not statistically significant. Importantly, the growing number of nonagenarians undergoing PCI may also reflect broader procedural eligibility as PCI has become safer and more feasible in very old patients. Thus, the temporal trend in mortality may be interpreted in the context of an increasingly inclusive PCI population, although the nonsignificant trend warrants further evaluation for procedural safety.

Chronological age should open the conversation rather than close it, and the benefit of each PCI procedure should be maximized on a case-by-case basis. Descriptive benchmarks of this kind are the substrate on which such individualized discussions rest, and incorporating frailty, functional status, and patient goals into future registry data collection may be a next step.
